# Comparison of Conscious and Deep Sedation Methods in Terms of Pulmonary Complications in ERCP Procedures of Patients with Billroth II Gastrectomy: A Retrospective Study

**DOI:** 10.3390/jcm14145099

**Published:** 2025-07-17

**Authors:** Ayse Lafci, Mehmet Sahap, Gokhan Erdem, Bulent Odemis

**Affiliations:** Ankara Bilkent City Hospital, Ankara 06800, Turkey; drsahap@gmail.com (M.S.); drgokhanerdem@gmail.com (G.E.); odemisbulentmd@yahoo.com (B.O.)

**Keywords:** Billroth II, endoscopic retrograde cholangiopancreatography, conscious sedation, deep sedation, pulmonary complications

## Abstract

**Background/Objective**: Patients who have undergone Billroth II gastrectomy may develop gastroparesis, hypomotility, and reflux esophagitis. These patients are at risk of aspiration of gastric contents into the lungs when subsequently sedated for Endoscopic Retrograde Cholangiopancreatography (ERCP) procedures. The aim of this study was to compare conscious sedation and deep sedation in terms of pulmonary complications in this selected cohort. **Methods**: Patients who had previously undergone Billroth II surgery and underwent ERCP procedure with sedation for gallstones or biliary tract strictures in a tertiary hospital between January 2020 and September 2023 were studied. Patient records were retrospectively obtained from the hospital information system. All the patients were divided into two groups as conscious sedation (Group CS) and deep sedation (Group DS). The groups were compared statistically in terms of pulmonary complications. **Results**: A total of 63 ERCP procedures were performed on 28 patients who had undergone Billroth II gastrectomy. There were 37 procedures involving conscious sedation (Group CS) and 26 involving deep sedation (Group DS). No statistically significant difference was found regarding pulmonary aspiration (*p* = 0.297) and other respiratory complications such as laryngospasm or desaturation between the two groups. In Group DS, it was observed that vomiting incidence was higher (*p* = 0.012), and airway maneuver requirements were increased (*p* = 0.007). **Conclusions**: In patients who have undergone Billroth II gastrectomy, both conscious sedation and deep sedation techniques can be used effectively during ERCP procedures. The complication rates and patient outcomes of the two techniques are comparable. The occurrence of respiratory complications leading to adverse post-procedural outcomes requires careful monitoring and meticulous follow-up for these patients.

## 1. Introduction

The management of anesthesia during ERCP procedures in patients with Billroth II gastrectomy involves several challenges due to anatomical changes and the shared airway between the endoscopist and the anesthesiologist during the procedure.

ERCP procedures can be performed under monitored anesthesia care with conscious sedation, deep sedation, or general anesthesia with endotracheal intubation [1,2]. However, ambulatory procedural sedation protocols are generally applied due to reasons such as patient admission costs and rapid patient recovery. In this rare but high-anesthesia-risk patient group, adequate sedation is necessary for the successful completion of the procedure. The depth of sedation in ERCP patients with Billroth II gastrectomy must be balanced between preventing pulmonary complications and ensuring procedural success.

Gastroparesis, hypomotility, remnant gastritis, and reflux esophagitis may occur after Billroth II surgery. In these patients, gastric pH increases due to the reflux of duodenal contents into the stomach. Studies have shown that widespread gastritis is observed in the remaining stomach after Billroth II [3]. In a study by Christodoulidis et al., bile reflux and alkaline gastritis were found in 54% of 74 patients who underwent Billroth II gastrectomy [4]. Pulmonary aspiration is defined as the presence of bile secretions or particulate matter in the tracheobronchial tree. It most commonly results from the passive regurgitation of gastric contents. In patients who have undergone Billroth II surgery and are sedated for ERCP, aspiration of gastric contents can lead to pneumonia due to decreased airway reflexes. In the literature, the frequency of aspiration during sedation has been reported to range between 0.01% and 1% [5,6]. Aspiration pneumonia is a significant cause of mortality.

During Billroth II surgery, Brown enteroenterostomy is an anastomosis performed between the afferent and efferent loops of the jejunum. Thanks to this procedure, gastritis, esophagitis, Barrett’s esophagus, and cancer formation can be prevented. If a Brown anastomosis is performed during Billroth II surgery, the risk of reflux and pulmonary aspiration may be lower [4].

There are many studies in the literature on anesthesia methods in ERCP procedures, but studies on sedation methods used for ERCP in patients who have undergone Billroth II surgery are limited. Our primary aim is to compare patients who underwent monitored anesthesia care with conscious sedation (Group CS) to those who underwent deep sedation (Group DS) among these selected patients, during procedures performed by the same experienced (with more than ten years of experience and over a thousand cases per year) endoscopist and anesthesia specialist, in terms of airway complications including pulmonary aspiration. Our secondary objectives are to investigate procedural success, intensive care unit (ICU) need, hospital stay duration, and mortality during hospitalization.

## 2. Materials and Methods

### 2.1. Ethical Approval

Ethical approval for this study was obtained from the Ethics Committee of Ankara City Hospital (Ankara, Turkiye) with the approval number E1-23-4134 on 18 October 2023. The study was conducted in accordance with the principles of the Declaration of Helsinki. Informed consent was waived due to the retrospective nature of the study.

### 2.2. Study Design and Setting

Between January 2020 and September 2023, patients who underwent ERCP procedures with conscious sedation and deep sedation in our hospital were retrospectively examined. Procedures performed by the same endoscopist and anesthesiologist within the specified period were included in the study. Procedures performed by different endoscopists, procedures in which the depth of sedation had to be changed during the same procedure, and procedures with incomplete records were excluded from the study. Some patients underwent the same procedure at different times with either conscious sedation or deep sedation; therefore, instead of the number of patients, the number of procedures was taken into account, and they were divided into Group CS and Group DS. The patients’ demographic data, comorbid conditions, and American Society of Anesthesiologists (ASA) scores were recorded.

All the patients underwent routine anesthesia monitoring (GE B125, GE Healthcare, Chicago, IL, USA) with pulse oximetry, electrocardiogram, and non-invasive blood pressure measurements at 2 min intervals. In Group CS, the patients were administered midazolam (2 to 10 mg i.v.) (Dormicum^®^, Roche, Basel, Switzerland) and fentanyl (0.05 to 0.1 mg i.v.) (Fentanyl Citrate^®^, Johnson & Johnson, New Brunswick, NJ, USA) titrated according to their age, accompanying diseases, and tolerance. In this group, the bispectral index (BIS) values of the patients were maintained in the range of 71–90.

In Group DS, after a bolus dose of propofol 0.5–1.0 mg/kg (Propofol^®^ 1%, Fresenius Kabi, Bad Homburg, Germany), the infusion dose was adjusted to 100–150 mcg/kg/min to maintain BIS values in the range of 61–70. All the patients were provided with oxygen support via nasal cannula (4 L/min). Apnea monitoring was performed with a capnograph. SpO2 below 90% for a duration of ten seconds or a decrease in oxygen saturation of more than 5% from the baseline value during anesthesia was defined as desaturation. Patients who experienced apnea or desaturation were given a jaw thrust maneuver to secure the airway. The stomach contents were examined using an endoscope, and oral aspiration was conducted at specific intervals; preparations for expedited endotracheal intubation were established in the event of a crisis. During the procedure, the patients who required intubation were recorded. A heart rate dropping below 50 beats/min was recorded as bradycardia, a heart rate exceeding 110 beats/min as tachycardia, a systolic blood pressure dropping below 90 mmHg or less than 85% of the baseline value as hypotension, and more than 20% above the baseline value as hypertension. The patients who were administered a vasopressor (5 mg ephedrine) due to hypotension during anesthesia were recorded. In the Billroth II surgery performed, those who had and had not undergone Brown anastomosis were recorded. Patient and endoscopist satisfaction were evaluated with “yes” and “no” answers to the questions. In the recovery phase, patients with an Aldrete score above 9 were considered to be fully recovered. Definite pulmonary aspiration symptoms were evaluated according to Bernardini et al.’s [7] criteria, which include dyspnea; hypoxia; auscultation abnormalities; aspiration of gastric contents, bile fluid, or other non-respiratory secretions from the trachea; and the presence of new infiltrates on chest X-ray. We adapted the criteria used by Ezri et al. [8] for possible pulmonary aspiration. These are as follows: the occurrence of laryngospasm or bronchospasm, admission to the intensive care unit due to anesthesia, and the continuation of mechanical ventilation after surgery were considered in cases of clinical suspicion of pulmonary aspiration without the detection of bile secretions or solid particles.

### 2.3. Statistical Analysis

The data analysis was conducted using the IBM SPSS 27.0 (IBM Corp, Armonk, NY, USA) statistical software package. While evaluating the study data, descriptive statistical methods (frequency, percentage, mean, standard deviation, median, and min-max) were used, and for the comparison of qualitative data, Pearson Chi-Square (χ^2^), Fisher’s exact χ^2^, or Yates’ χ^2^ tests were used as appropriate. The normality of the data was evaluated using the Kolmogorov–Smirnov test, skewness–kurtosis, and graphical methods (histogram, Q-Q plot, Stem-and-Leaf, and Boxplot). In the study, the Independent Samples *t*-test (*t*-test for independent groups) was used to evaluate quantitative data showing a normal distribution. The statistical significance level was accepted as *p* < 0.05.

Sample Size Calculation: (Calculated according to the need for airway maneuver) Power analysis was conducted using the G* Power 3.1.9.7 (Franz Faul, University of Kiel, Kiel, Germany) statistical software package; n1 = 26 (P1 = 0.24), n2 = 37 (P2 = 0.62), OR = 5.12, and α = 0.05, resulting in a power of 84%.

## 3. Results

A total of 63 ERCP procedures were performed on 28 patients who had undergone Billroth II gastrectomy. Of these, 37 procedures were performed under conscious sedation (Group CS), and 26 procedures under deep sedation (Group DS). Thirteen patients received only conscious sedation during repeated procedures, while 11 received only deep sedation. Four patients underwent some procedures with conscious sedation and others with deep sedation.

The average age of the patients included in the study was 72.6 ± 10.4 years, with 7 women and 21 men. The average body mass index (BMI) was 20.3 ± 1.9 (Table 1).

Considering the procedures, 24 (38.1%) involved ASA II patients, and 39 (61.9%) involved ASA III patients. Among these procedures, comorbidities included 34.9% diabetes mellitus (DM), 71.4% hypertension (HT), 41.3% chronic heart disease, 23.8% chronic kidney disease, and 14.3% chronic pulmonary disease.

When comparing according to the type of anesthesia, it was found that in Group DS, the rate of chronic kidney disease was lower, the number of ASA II patients was higher, anesthesia duration was shorter, recovery time was longer, the vomiting rate was higher, and the need for airway maneuvers was greater. There was no statistically significant difference between the groups in terms of other variables (*p* > 0.05).

No statistically significant difference was found between Group CS and Group DS regarding pulmonary aspiration and other respiratory complications. Only in the deep sedation group, one patient developed a ruptured hydatid cyst followed by pulmonary aspiration, requiring post-procedural mechanical ventilation, which resulted in mortality. The requirements for non-invasive mechanical ventilation (Group CS 2.7%, Group DS 7.7%, *p* = 0.564) and oxygen via mask (Group CS 10.8%, Group DS 19.2%, *p* = 0.469) were similar between the groups (Table 2).

**Figure 1:**
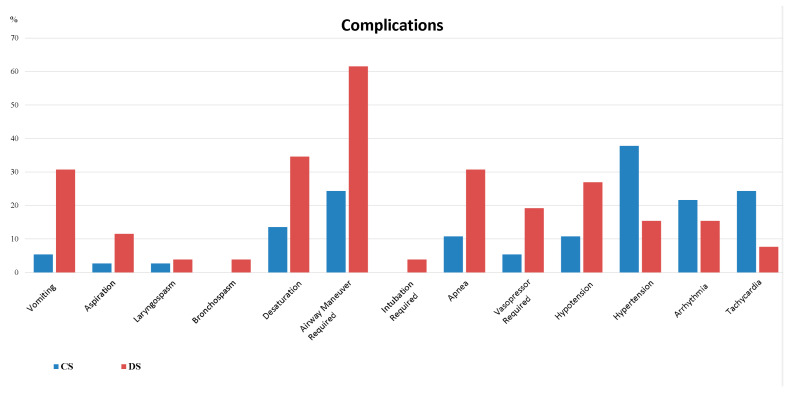
Comparison of complications between groups. CS = conscious sedation; DS = deep sedation.

**Figure 2:**
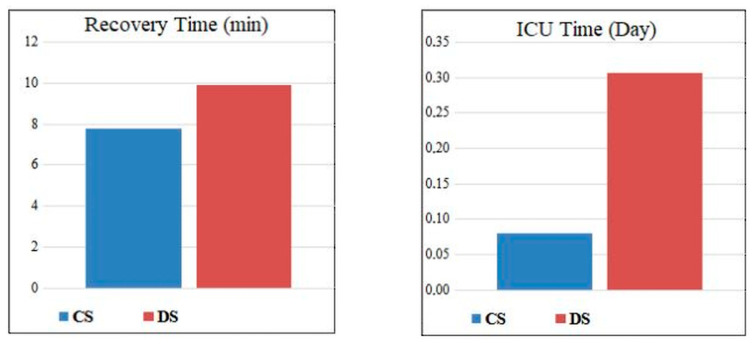
Comparison of recovery and intensive care hospitalization days of the groups. CS = conscious sedation; DS = deep sedation.

In the post-procedural intergroup comparison, similar rates of pulmonary infiltrate (Group CS 2.7%, Group DS 3.8%, *p* = 1.000), patient satisfaction, and endoscopist satisfaction were observed. The need for intensive care (Group CS 2.7%, Group DS 3.8%, *p* = 1.000) and the duration of intensive care stay were also similar between the groups (Table 3, Figure 1 and Figure 2).

## 4. Discussion

In patients who have undergone Billroth II gastrectomy and subsequently underwent ERCP performed by the same experienced endoscopist and anesthesiologist, we observed that conscious sedation and deep sedation were comparable in terms of pulmonary aspiration and other respiratory complications.

Contrary to our initial expectation of a lower aspiration rate in the conscious sedation group, the rates of aspiration were statistically similar between the two groups. Aspiration occurred in three patients in the deep sedation group (Group DS) and in one patient in the conscious sedation group (Group CS). The relatively small sample size likely contributed to the lack of statistical significance. In the conscious sedation group, midazolam use may have promoted reflux and aspiration by causing relaxation of the lower esophageal sphincter [9]. Conversely, propofol—used for deep sedation—is known to have a lower emetogenic potential and acts as a potent 5-HT3 receptor blocker [10]. Its antiemetic effects may be attributed to direct inhibition of the chemoreceptor trigger zone, vagal nuclei, and other central pathways involved in nausea and vomiting [11]. These pharmacological properties could have contributed to the similar incidence of vomiting and pulmonary aspiration observed in the deep sedation group.

Some studies suggest that superficial sedation with propofol administered by the endoscopist results in fewer side effects compared to deep sedation performed by an anesthesiologist [12]. However, an experienced anesthesiologist is better equipped to manage and intervene in sedation-related complications effectively. Unlike the study by Poincloux et al., which excluded patients with significant comorbidities, our cohort included elderly patients with multiple comorbid conditions, such as diabetes mellitus, hypertension, chronic heart disease, chronic kidney disease, and chronic pulmonary disease. These factors inherently increase the risk of aspiration, particularly in diabetic patients with autonomic neuropathy. This was present in nine diabetic patients undergoing 22 ERCP procedures in our study.

A review by Gareval et al. supports deep sedation with propofol as a superior technique to conscious sedation for ERCP, citing better recovery profiles and lower failure rates, albeit emphasizing that such sedation should be administered or supervised by an anesthetist [13]. In our study, anesthesia-related complications were low and comparable between groups. Although vomiting was more frequent in the deep sedation group, the need for airway interventions, aspiration, laryngospasm, bronchospasm, apnea, and intubation did not differ significantly. We attribute this safety anesthesia to our use of bispectral index (BIS) monitoring for titrating sedation depth. Park et al. demonstrated through a systematic meta-analysis that BIS monitoring during endoscopic procedures effectively prevents excessive propofol dosing while maintaining adequate sedation [14]. Similarly, clinical studies confirm a strong correlation between BIS values and clinical sedation scales [15]. Thus, BIS monitoring likely helped balance anesthetic safety and efficacy in our patients.

Airway maneuvers such as chin lifting were sufficient in most cases. Consistent with a systematic review and meta-analysis by Dhaliwal et al. on sedation preferences for ERCP, anesthesia durations were longer in the conscious sedation group, likely because procedure performance is more challenging in conscious patients [16]. Conversely, recovery times were longer in the deep sedation group, but this did not prolong respiratory support post-procedure.

Patients undergoing Billroth II surgery have stomach malignancies and related nutritional problems. Consequently, their BMI values are low. The patients’ low body mass index (BMI) in our study underscores a scenario that necessitates prudence in anesthetic strategies. It is recognized that heightened susceptibility to hypnotic drugs like propofol in patients with a low BMI can elevate the risk of respiratory and hemodynamic problems during severe sedation. In our investigation, sedation operations were conducted exclusively by anesthesiologists proficient in this domain; dosages were tailored and delivered by titration. The hypotension experienced by the patients was transient and brief, necessitating no intervention. The arrhythmias observed were sinus arrhythmias, and they returned to normal sinus rhythm without intervention after the procedure.

A serious adverse event occurred in a patient undergoing deep sedation ERCP for cholangitis, who experienced a ruptured hydatid cyst and subsequent pulmonary aspiration, leading to mechanical ventilation and eventual mortality. Although mortality did not statistically differ between groups, this complication highlights the critical importance of vigilance by both anesthetists and endoscopists.

Technical success rates were similar between the groups. Key procedural steps—such as accessing the afferent loop, the papilla, and successful cannulation—were not impeded by sedation type, although altered anatomy after Billroth II surgery inherently complicates ERCP procedures [17]. Only one procedure failure occurred, unrelated to sedation depth, underscoring the importance of the procedure being performed by an experienced endoscopist.

Brown enteroenterostomy performed during Billroth II surgery is known to reduce the risk of gastritis, esophagitis, reflux, and aspiration. In our cohort, Brown anastomosis was present in a small number of procedures (five in Group CS and four in Group DS), and sedation type did not influence complication rates in these patients.

## 5. Limitations

The limitations of this study, in addition to being a retrospective study, were due to the relatively limited number of procedures because it involved a selected patient group. Despite being a center where a high number of ERCP procedures are performed, the relatively low number of ERCP procedures in patients who underwent Billroth II surgery did not sufficiently reflect in our statistical results. Further studies with a larger number of patients are needed in this regard.

## 6. Conclusions

In patients who have undergone Billroth II gastrectomy, both conscious sedation and deep sedation methods are safe and effective for ERCP procedures. There is no significant difference between the two methods in terms of complication rates, patient and endoscopist satisfaction, and clinical outcomes. The interrelatedness of respiratory complications and their contribution to poor post-procedural outcomes underscores the importance of careful monitoring, close follow-up, and early intervention in these patients.

## Figures and Tables

**Table 1:** Characteristics of the patients.

		*n* Mean ± SD	% Median (Min–Max)
Gender ^a^	Female	7	11.1
	Male	21	33.3
Age (year) ^b^		72.6 ± 10.4	74.0 (44.0–88.0)
BMI (kg/m^2^) ^b^		20.3 ± 1.9	20.1 (16.7–24.1)
Number of Procedures ^b^		2.3 ± 1.4	2.0 (1.0–5.0)
1 ^a^		11	17.5
2 ^a^		8	12.7
3 ^a^		3	4.8
4 ^a^		3	4.8
5 ^a^		3	4.8

^a^: n/%, ^b^: mean ± SD/median (Min–Max); BMI = body mass index; SD = standard deviation.

**Table 2:** Comparison of groups according to type of anesthesia.

		Anesthesia Type		*p*
		Group CS	Group DS	
		(*n* = 37)	(*n* = 26)	
Comorbidities	Diabetes Mellitus	15 (40.5%)	7 (26.9%)	0.397 ^a^
	Hypertension	28 (75.7%)	17 (65.4%)	0.544 ^a^
	Chronic Heart Disease	17 (45.9%)	9 (34.6%)	0.523 ^a^
	Chronic Kidney Disease	13 (35.1%)	2 (7.7%)	**0.027** ^a^
	Chronic Pulmoner Disease	8 (21.6%)	1 (3.8%)	0.069 ^a^
ASA	II	9 (24.3%)	15 (57.7%)	**0.015** ^a^
	III	28 (75.7%)	11 (42.3%)	
Anesthesia Time (min)		44.6 ± 8.6	39.4 ± 10.5	**0.035** ^b^
Procedure Time (min)		40.5 ± 9.0	36.0 ± 10.6	0.074 ^b^
Recovery Time (min)		7.8 ± 2.3	9.9 ± 1.5	**<0.001** ^b^
Brown Anastomosis	Absent	32 (86.5%)	22 (84.6%)	1.000 ^a^
	Present	5 (13.5%)	4 (15.4%)	
Complications	Vomiting	2 (5.4%)	8 (30.8%)	**0.012** ^a^
	Aspiration	1 (2.7%)	3 (11.5%)	0.297 ^a^
	Laryngospasm	1 (2.7%)	1 (3.8%)	1.000 ^a^
	Bronchospasm	0 (0.0%)	1 (3.8%)	0.413 ^a^
	Desaturation	5 (13.5%)	9 (34.6%)	0.094 ^a^
	Airway Maneuver Required	9 (24.3%)	16 (61.5%)	**0.007** ^a^
	Intubation Required	0 (0.0%)	1 (3.8%)	0.413 ^a^
	Apnea	4 (10.8%)	8 (30.8%)	0.058 ^a^
	Vasopressor Required	2 (5.4%)	5 (19.2%)	0.114 ^a^
	Hypotension	4 (10.8%)	7 (26.9%)	0.176 ^a^
	Hypertension	14 (37.8%)	4 (15.4%)	0.097 ^a^
	Arrhythmia	8 (21.6%)	4 (15.4%)	0.746 ^a^
	Tachycardia	9 (24.3%)	2 (7.7%)	0.105 ^a^
Procedure Termination	Absent	36 (97.3%)	25 (96.2%)	1.000 ^a^
	Present	1 (2.7%)	1 (3.8%)	

^a^: Chi-Square Test (n (%)), ^b^: Independent Samples *t*-test (Mean ± SD), ASA = American Society of Anesthesiologists; CS = conscious sedation; DS = deep sedation. Bold values are *p* < 0.05 and are considered statistically significant.

**Table 3:** Post-procedural comparison by anesthesia type.

		Anesthesia Type		*p*
		CS	DS	
		(*n* = 37)	(*n* = 26)	
Postop Admission Unit	WARD	36 (97.3%)	25 (96.2%)	1.000 ^a^
	Intensive Care Unit	1 (2.7%)	1 (3.8%)	
Intensive Care Unit Time (day)		0.1 ± 0.5	0.3 ± 1.6	0.413 ^b^
	0 Day	36 (97.3%)	25 (96.2%)	0.345 ^a^
	3 Day	1 (2.7%)	0 (0.0%)	
	8 Day	0 (0.0%)	1 (3.8%)	
Hospital Stay (days)		3.6 ± 3.1	2.7 ± 3.3	0.269 ^b^
Breating	Mechanical Ventilation Required	0 (0.0%)	1 (3.8%)	0.413 ^a^
	Non-Invasive Ventilation	1 (2.7%)	2 (7.7%)	0.564 ^a^
	Oxygen by Mask	4 (10.8%)	5 (19.2%)	0.469 ^a^
Infiltration on Chest X-ray	Absent	36 (97.3%)	25 (96.2%)	1.000 ^a^
	Present	1 (2.7%)	1 (3.8%)	
Patient Satisfaction	No	1 (2.7%)	1 (3.8%)	1.000 ^a^
	Yes	36 (97.3%)	25 (96.2%)	
Endoscopist Satisfaction	No	2 (5.4%)	1 (3.8%)	1.000 ^a^
	Yes	35 (94.6%)	25 (96.2%)	
Mortality	No	37 (100.0%)	25 (96.2%)	0.413 ^a^
	Yes	0 (0.0%)	1 (3.8%)	

^a^: Chi-Square Test (n (%)), ^b^: Independent Samples *t*-test (Mean ± SD).

## Data Availability

The original contributions presented in this study are included in the article. Further inquiries can be directed to the corresponding author(s).
